# Radiosensitisation of U87MG brain tumours by anti-epidermal growth factor receptor monoclonal antibodies

**DOI:** 10.1038/sj.bjc.6604943

**Published:** 2009-03-17

**Authors:** A Diaz Miqueli, J Rolff, M Lemm, I Fichtner, R Perez, E Montero

**Affiliations:** 1 Department of Experimental Immunotherapy, Centre of Molecular Immunology, P.O. Box 16040, 214 Street, Havana 11600, Cuba; 2 Department of Experimental Pharmacology, Max Delbrück Center for Molecular Medicine. Robert-Rössle-Strasse 10, Berlin-Buch 13125, Germany

**Keywords:** CD133, cetuximab, epidermal growth factor receptor, glioblastoma multiforme, nimotuzumab, radiation.

## Abstract

As epidermal growth factor receptor (EGFR) has been reported to be a radiation response modulator, HER inhibitors are regarded to act as potential radiosensitisers. Our study examined the role of nimotuzumab and cetuximab both, the two monoclonal antibodies (mAbs) to EGFR, as radiosensitisers in a murine glioma model *in vivo*. Co-administration of both the antibodies with radiation increased the radiosensitivity of U87MG, resulting in a significant delay of subcutaneous (s.c.) tumour growth. Furthermore, the addition of antibodies to the radiation decreased brain tumour sizes and is inhibited by 40–80% the increased tumour cell invasion provoked by radiotherapy, although promoted tumour cell apoptosis. Whereas nimotuzumab led to a reduction in the size of tumour blood vessels and proliferating cells in s.c. tumours, cetuximab had no significant antiangiogenic nor antiproliferative activity. In contrast, cetuximab induced a more marked inhibition of EGFR downstream signalling compared with nimotuzumab. Moreover, both antibodies reduced the total number of radioresistant CD133+ cancer stem cells (CSCs). These results were encouraging, and showed the superiority of combined treatment of mAbs to EGFR and radiation over each single therapy against glioblastoma multiforme (GBM), confirming the role of these drugs as radiosensitisers in human GBM. In addition, we first showed the ability of mAb specifics against EGFR to target radioresistant glioma CSC, supporting the potential use in patients.

Glioblastoma multiforme (GBM) remains a deadly disease with a median survival of <1 year despite all therapeutic efforts based on the aggressive conventional anticancer therapies (Quigley *et al*, 2007). An attempt to improve local control of the disease and overall survival of patients with GBM has considered the possibility to add newer forms of treatment with conventional therapies such as chemo- and radiotherapy (Hauch *et al*, 2005).

A well-known validated target for molecular anticancer-targeted therapies is the epidermal growth factor receptor (EGFR) (Prenzel *et al*, 2001). There is strong evidence in support of this: EGFR is frequently overexpressed in ∼40% of GBMs, its overexpression correlates with a poor prognosis in patients, and early studies using specific anti-EGFR agents have shown efficacy to increase survival in mice xenotransplanted with tumours directly established from patients (Eller *et al*, 2002). Indeed, several of these agents have entered its evaluation in patients given alone or in combination with conventional therapies (e.g. radiotherapy). In addition, such studies have correlated the overexpression of the EGFR with radioresistance (Barker *et al*, 2001; Bonner *et al*, 2004), whereas the EGFR blockade increases the sensitivity of tumour cells to radiation therapy (Huang and Harari, 2000).

Among EGFR inhibitors, nimotuzumab (TheraCIM h-R3) and cetuximab (IMC-C225) have undergone an extensive evaluation in different tumour locations, showing activity when administered with ionising radiation (Bonner *et al*, 2004; Crombet *et al*, 2004; Ramos *et al*, 2006). However, these studies did not dissect comparatively the contribution of individual therapies for such antitumour effect. To investigate the advantages resulting from the addition of anti-EGFR antibodies to the radiation therapy over a single-agent therapy, we investigated the growth-inhibitory effects of both nimotuzumab and cetuximab, alone and in combination with radiation in a human GBM xenografted in NMRI nude mice. We also explored potential mechanisms of such antitumour activity underlying the therapeutic effect.

## Materials and methods

### Cell culture

U87MG (ATCC HTB-14, Rockville, MD, USA) is a human GBM cell line. Cells were grown in a 1+1 mixture of Eagle's minimum essential medium and Basal medium, (Sigma, St Louis, MO, USA) containing 2 mM L-glutamine and 10% foetal bovine serum, and were maintained under a humidified atmosphere of 5% CO_2_ at 37°C.

### Antibodies

The human–mouse chimeric anti-EGFR monoclonal antibody (mAb) cetuximab was provided by ImClone Systems, Inc. (New York, NY, USA). The humanised anti-EGFR mAb nimotuzumab was generated at the Centre of Molecular Immunology (Mateo *et al*, 1997). All the primary and secondary antibodies were purchased from commercial sources listed as follows: rabbit polyclonal EGFR antibody to total EGFR (Santa Cruz Biotechnology Inc., Santa Cruz, CA, USA), mouse monoclonal anti-phospho-tyrosine antibody (BD Pharmingen, San Diego, CA, USA), mouse monoclonal ERK1/2 antibody to total ERK1/2 (BD Pharmingen), rabbit polyclonal phospho-p44/42 MAPK (Thr202/Tyr204) antibody to activated ERK1/2 (BD Pharmingen), mouse monoclonal antibody MIB-1 to Ki-67 (DakoCytomation, Carpinteria, CA, USA), rat monoclonal anti CD31/PECAM-1 antibody (BD Pharmingen), biotin-conjugated mouse monoclonal antibody to CD133/1 (AC133) (Miltenyi Biotec, Cologne, Germany). The secondary antibodies used were as follows: horseradish peroxidase (HRP)-conjugated anti-rat IgG1 (Southern Biotech, Birmingham, AL, USA), HRP-conjugated anti-rabbit IgG (DakoCytomation) and HRP-conjugated anti-mouse IgG (DakoCytomation).

### Proliferation index (Ki-67), angiogenesis (CD31/PECAM-1), apoptosis (TUNEL) and immunohistochemistry

Until analysis all the specimens were shock frozen and stored in nitrogen. Immunostaining was performed using 5-*μ*m tissue sections placed on glass slides. The proliferation index was calculated from tissue sections stained using an indirect immunoperoxidase technique that employed the biotinylated clone MIB-1 (1 : 50 dilution) as primary antibody, which is specific for the Ki-67 protein, a marker of proliferating cells, followed by incubation with streptavidine/HRP (DakoCytomation). At least 5 high-power fields were counted and scored per sample. To score a tumour cell as positive for Ki-67, a nuclear staining was required. For this methodology, tumour slices were fixed with Fix I solution (NKH (8 g l^−1^ NaCl and 0.4 g l^−1^ KC1 buffered with 0.1 M Hepes, pH 7.4), 1 M HEPES, 25% glutaraldehyde, 1% glucose). All other immunohistochemical stains were performed on paraformaldehyde-fixed, cryostat 5-*μ*m tissue sections using standard techniques. The following primary antibodies were used: anti-human CD133/1 (1 : 10 dilution), anti-human EGFR (1 : 500 dilution) and anti CD31/PECAM-1 (1 : 100 dilution). After washing, corresponding HRP-conjugated secondary antibody were used to detect antibody–antigen reactions. The negative controls consisted of duplicate sections of the same specimens, in which the primary antibody had been excluded and replaced with PBS or negative control immunoglobulin. The sections were visualised with 3,3′-diaminobenzidine as a chromogen and counterstained with Mayer's hematoxylin. Representative tumour sections were identified on a light microscope (Zeiss, Axioskop 40) with an ocular magnification of × 40 evaluating 4–5 tumours from each group for the corresponding analysis. To score a tumour cell as positive, a complete membrane staining was required for EGFR and CD133. Total area of the endothelial blood microvessels was calculated using the software (Axio Vison 4.5). Fluorescent terminal-deoxynucleotidyl-transferase-mediated nick end labelling (TUNEL) to evaluate apoptosis was done as per the manufacturer's instructions (Roche Diagnostics, Mannheim, Germany). Slides were analysed in a drop of PBS with a fluorescence microscope (Zeiss, Axioskop 40) in five random fields at × 40 magnification.

### Western blotting

Western blotting experiments were performed as described (Diaz *et al*, 2007). Briefly, tumour lysates were fractioned by sodium dodecyl sulphate–polyacrylamide gel electrophoresis on a 7.5% gel, and the separated proteins were transferred to a nitrocellulose membrane. After blocking of non-specific sites, the membrane was incubated with primary antibodies to activated EGFR (1 : 500 dilution), total ERK1/2 (1 : 500 dilution), activated ERK1/2 (1 : 1000 dilution) and total EGFR (1 : 500 dilution). Subsequently, membranes were incubated with the corresponding HRP-conjugated secondary antibody. Bound antibody was visualised using a western blotting chemiluminescence luminol reagent system (Santa Cruz Biotechnology).

### Animal experiments

Female athymic mice (8–10 weeks old, *nu/nu*) were obtained from Charles River (Sulzfeld, Germany). The mice were housed and maintained under aseptic conditions in facilities approved by the German Association for Accreditation of Laboratory Animal Care and in accordance with current regulations and standards of the German Animal Protection Law, and their use was approved by the local responsible authorities. Animals met the requirements of the UKCCCR guidelines (1998). To produce xenografts, tumour cells were harvested from subconfluent cultures by treatment with 0.25% trypsin and 0.05% EDTA. Only single-cell suspensions with >90% viability were used for injections. Animals were inoculated with both 10^7^ U87MG tumour cells subcutaneous (s.c.) into left flank and 2 × 10^4^ cells intracranially into the right hemisphere of mouse brains with the help of a stereotactic device. Tumour volume from flank's were determined from direct measurement with callipers and calculated according to the formula as follows: 0.5 × (large diameter) × (small diameter)^2^. Relative tumour volumes (RTV) were calculated by referring the median volumes of each day to the first measurement (set to 1) as described (Mansour *et al*, 2003). Treatments were initiated 3 days after tumour cell injection. Treatment groups consisted of control, nimotuzumab or cetuximab alone, radiation alone, and the combination of each antibody and radiation, with each group containing eight mice, except the control group containing ten mice. Both antibodies were administered intraperitoneal three times per week with 1 mg per mouse, (50 mg kg^−1^) mice in the control and radiation-alone groups were injected with saline solution. For radiation groups, animals were exposed to a total dose of 3.0 Gy of total body radiation fractioned in 1.0 Gy weekly. In the combination group the antibodies were administered 6 h before radiation therapy. All animals were killed by day 28 when tumour weight from the control group exceeded the 10% of total animal weight. These studies were repeated twice both for subcutaneous and intracranial tumours.

The size of intracranial tumours was determined. For that purpose, brains were collected and shock frozen in 2-methylbutane. Sequential cryosections (10 *μ*m) were prepared and stained with cresyl violet. At the area of largest dimensions tumour diameters and perimeters were determined by computer-assisted image with the help of a microscope (Zeiss Axioskop 40). Subcutaneous tumours were snapping frozen and stored at −80°C for additional analyses.

### Statistical analysis

Statistical analysis was performed using the GraphPAD Prism software for Windows, version 4.0 (GraphPAD, San Diego, CA, USA). The significance of differences between groups was compared using a Kruskal–Wallis test. The significance of differences in groups was compared using Dunn′s multiple comparison test. Differences were considered significant if *P*<0.05.

## Results

### Radiosensitisation of U87MG tumours xenografted subcutaneously *in vivo* with anti-EGFR mAb

Human U87MG GBM cell line sensitivity to radiation is influenced by the experimental conditions (Suit *et al*, 1990; Eshleman *et al*, 2002), allowing to evidence combinatorial effects when used concomitantly to other therapeutic approaches (Wachsberger *et al*, 2007).

**Figure 1:**
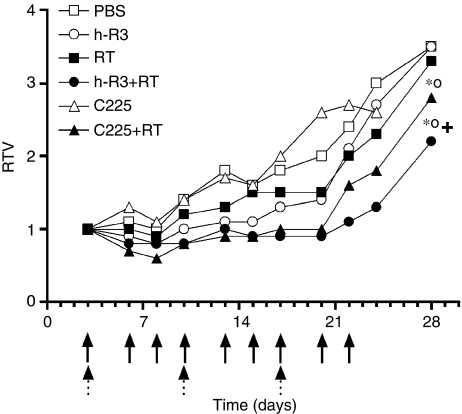
Sensitization of U87MG human tumour subcutaneously xenografted into NMRI nude mice to radiation by anti-EGFR mAb. Cells were injected subcutaneously in athymic mice. Treatments were initiated 3 days after tumour inoculation with ○ nimotuzumab (h-R3), or ▵ cetuximab (C225), 50 mg kg^−1^ intraperitoneally, three times per week by 3 weeks, or ▪ radiation (RT), 3 Gy fractioned in 1 Gy weekly, or both modalities • (h-R3+RT), ▴ (C225+RT) or □ PBS control (PBS). Antibody administrations are showed as black arrows and radiation as fractioned arrows. Tumour volume was determined at the indicated time thereafter and RTV were calculated. Error bars are not shown because of better clarity. Kruskal–Wallis test; symbols indicate statistical differences as follows: ^*^Significant to PBS, +significant to antibody alone, °significant to radiation.

Here, we explored the combinatorial effect of the EGFR targeted therapy based on mAbs with radiotherapy. U87MG cell line was xenografted both in the flanks and in the brains of NMRI nude mice. All animals injected with the tumour cells developed tumours. The mice were then treated with either nimotuzumab or cetuximab, radiation or both modalities during 3 weeks. Figure 1 shows the RTV of treated groups at different time points. Combined treatment with radiation and antibodies resulted in a substantial growth delay and subsequent inhibition of the growth rate of U87MG xenografts with a maximum effect by day 28, that is: media RTV for h-R3+RT (2.2) and C255+RT (2.8) *vs* control (3.5). In contrast, radiation and antibodies each as a single modality treatment did not affect tumour growth profiles compared with controls. Further, no significant decreases in body weight were observed in mice in any of the six treatment groups. These results suggest that nimotuzumab and cetuximab may enhance the antitumour activity of radiation in U87MG tumours *in vivo*.

### Inhibition of tumour invasion promoted by radiation in U87MG tumours *in vivo* by anti-EGFR mAb

**Figure 2:**
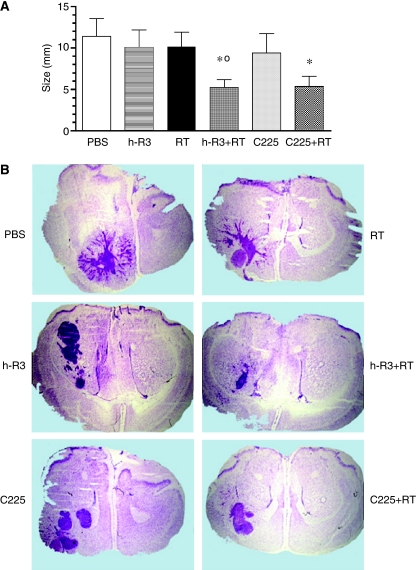
Sensitization of U87MG human tumour orthotopically xenografted into NMRI nude mice to radiation by the anti-EGFR mAb. (**A**) Cells were injected intracranially in athymic mice. Treatments were initiated 3 days after tumour inoculation. The antibody was administered at 50 mg kg^−1^ intraperitoneally, three times per weeks by 3 weeks. Animals receiving radiation were exposed to a total dose of 3 Gy fractioned in 1 Gy weekly. (**B**) Stained sections show the extent and morphology of tumours treated with PBS control (PBS), radiation alone (RT), nimotuzumab alone (h-R3), or cetuximab alone (C225), or both modalities. Analysed brain sections from mice showed a remarkable reduction in the number of small satellite tumours in the groups of mice treated with the antibodies alone or in combination with radiation. Kruskal–Wallis test; symbols indicate statistical differences as follows: ^*^Significant to PBS, °significant to radiation.

To reproduce a closer real life situation, we examined whether these antibodies may enhance the antitumour activity of radiation in U87MG tumours implanted in the brain of athymic mice. In the orthotopic model (Figure 2A), the radiation failed to show a statistical reduction in brain tumour size (*P*>0.05), as well as each antibody given alone. In contrast, only the combination of each antibody with radiation reduced significantly (*P*<0.05) the growth of orthotopic tumours; for example: tumour perimeter (mean±s.e.m.) for h-R3+RT (5.2±0.9) and C255+RT (5.4±1.2) *versus* control (11.1±2.1). More interesting, a histopathological analysis of intracranial tumour sections showed a strikingly more invasive growth pattern in mice treated with radiotherapy alone compared with antibodies-based therapies. These tumours were usually surrounded by numerous small-satellite tumours (Figure 2B). Quantification of these satellites showed that the satellite frequency (median, min–max) was increased over 40% in mice exposed to radiotherapy (26, 11–51) compared with control (18, 3–40). Contrastingly, in mice receiving antibodies-based therapies a 40–80% of reduction in the number of satellite tumours was documented; that is: h-R3+RT (9, 1–25) and C255+RT (4, 0–17). Interestingly, monotherapy with both antibodies also display a reduction in the frequency of satellite tumour; that is: h-R3 (10, 0–24) and C255 (10, 0–26) (Supplementary Table 1). These results suggest that both antibodies may increase the radiosensitisation of U87MG tumours in the brain of mice, whereas decrease the satellite tumour formation induced by radiation.

### Radiosensitisation of U87MG tumours by anti-EGFR mAb *in vivo* occurred by different mechanisms

**Figure 3:**
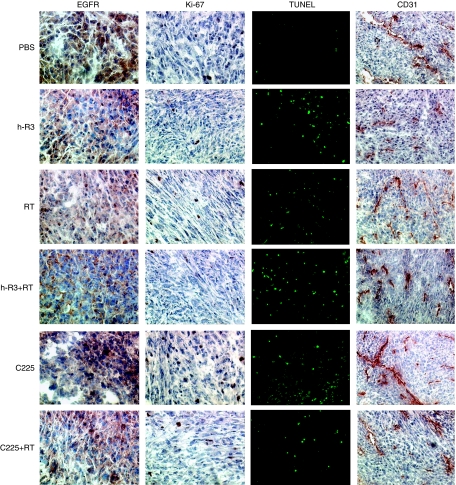
Tissue-based studies of U87MG human tumours xenografted into NMRI nude mice treated with nimotuzumab (h-R3), or cetuximab (C225), or radiation alone (RT), or both modalities. Immunohistochemical analysis of tumour cells stained with anti-EGFR, anti-Ki-67 nuclear antigen, apoptosis by TUNEL and angiogenesis with anti-CD31 antibody ( × 40 magnification).

To evaluate mechanisms underlying the antitumour effect described above, an immunohistochemical analysis was done at the end of the treatment in tumour specimens excised from the s.c. area. A positive EGFR immunostaining was detected in all analysed tumour samples (Figure 3). Furthermore, data scored from +1 to +4 as per immunostaining intensity was blinded evaluated resulting nearly identical in each treatment group, indicating no differences in the EGFR expression level in analysed tumours (Data not shown). EGFR expression was also verified by western blot analysis, showing similar results (Supplementary Figure 1).

**Figure 4:**
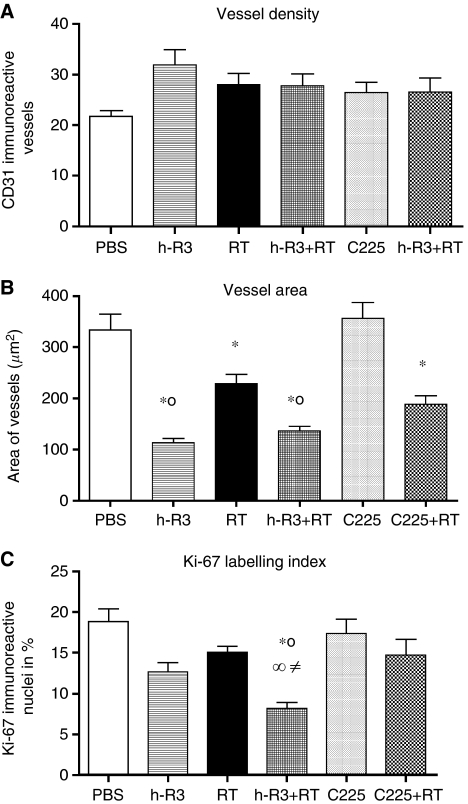
Comparisons between tumours treated with PBS control (PBS), or nimotuzumab (h-R3), or cetuximab (C225), or radiation alone (RT), or both modalities. (**A**, **B**) Angiogenesis by vessel density and vessel area. (**C**) Proliferation index by Ki-67. Kruskal–Wallis test; symbols indicate statistical differences as follows: ^*^Significant to PBS (*P*<0.001), ^∞^significant to cetuximab alone (*P*<0.001), °significant to radiation (*P*<0.01), ^≠^significant to cetuximab plus radiation (*P*<0.05).

Given that angiogenesis is considered a process of neovascularisation particularly pertinent in gliomas that permits malignant cells spread diffusely as the brain is a highly vascularised organ, we evaluated whether both antibodies might inhibits angiogenic processes in this glioma model. A quantitative analysis of the blood vessels stained with the specific endothelial marker CD31 did not show differences in the microvessel density of s.c. tumours (Figure 4A), but showed striking differences in the size of the vascular channels (expressed as median in *μ*m±s.e.m.), among different group of treatments (Figures 3 and 4B). Whereas tumours in mice treated with nimotuzumab, exhibited a significant reduction (two-folds) in the vessel size (113.4±8.2), many enlarged vessels were found in control mice (333.3±31.6) or treated with radiation alone (228.3±18.6). These mice exhibited highly enlarged vessels forming complex structures suggestive of splitting of the vessel lumen by endothelial intravessel walls (Figure 3). Furthermore, similar reductions in the size of blood vessels after treatment with nimotuzumab alone, or both nimotuzumab and radiation (135.8±10.1), suggested that the antiangiogenic activity was mainly associated to the use of the antibody. These results indicate that no higher antiangiogenic potential between radiation and the antibody for the doses chosen could be expected; despite the antitumour activity observed with both therapies seems to be higher than expected by independent treatments. In contrast, in mice treated with cetuximab, similar reductions in the size of the vessels were observed only when the antibody was added to the radiotherapy (188.3±16.5), but it was mainly attributable to the effect of the radiation, indicating no significant antiangiogenic activity for cetuximab.

Antiangiogenic and pro-apoptotic processes are commonly linked to the activity of the anti-EGFR agents. Subsequently, spontaneous apoptosis (TUNEL) in the control group was negligible, sharply contrasting with the different groups of treatment; however, no statistical differences between them were found (Figure 3).

We next examined whether both antibodies might enhance the antiproliferative effect of radiation by analysing the Ki-67 expression. The analysis of the proliferative activity in these tumours showed that, nimotuzumab enhanced the inhibition of proliferation caused by radiation by 56.5% in the combination group, compared with the antibody alone or radiotherapy (39.9 or 19.9%, respectively; Figures 3 and 4C). These results indicate an antiproliferative effect of nimotuzumab alone that may by further augmented when combined to radiation. Cetuximab in contrast, did not produce a significant inhibition on cell proliferation neither alone (7.8%), nor added to radiotherapy (21.7%). Moreover, a statistical reduction (*P*<0.05) on cell proliferation was reached between the two groups of combination.

### Enhanced inhibition of EGFR signalling in U87MG tumours by anti-EGFR mAb *in vivo*

**Figure 5:**
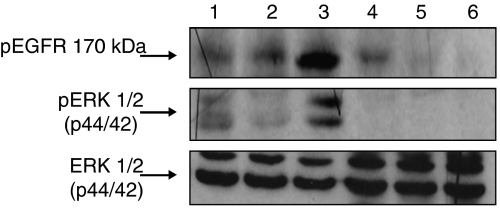
EGFR and ERK1/2 phosphorylation in U87MG tumours xenografted into NMRI nude mice after treatment with nimotuzumab, or cetuximab, or radiation alone, or both modalities. Immunoblot analysis of (upper panel) tyrosine phosphorylated EGFR, or (middle panel) activated ERK1/2, or (bottom panel) ERK1/2 protein expression in tumours. Lane 1 PBS control, lane 2 nimotuzumab, lane 3 radiation, lane 4 nimotuzumab plus radiation, lane 5 cetuximab, lane 6 cetuximab plus radiation. Immunoblots were developed using chemiluminescence. Abbreviations: pEGFR, receptor tyrosine phosphorylation; ERK1/2, Extracellular signal-regulated kinases 1 and 2. Pictures show one representative example per group out of four samples analysed.

To further examine potential advantages of the combined therapy over a single-drug treatment, we determined the effect of different treatments on EGFR signalling by western blotting. Constitutive activation of EGFR was unaffected in mice treated with nimotuzumab, although it increased after radiation alone (Figure 5, upper panel). In contrast, a more pronounced decrease in the phosphorylation of ERK1/2 proteins was observed after treatment with nimotuzumab and radiation, compared with each single therapy (Figure 5, middle panel), indicating that the inhibition of EGFR signalling by nimotuzumab may increase by using the antibody in addition to radiation, consistent with the inhibitory-growth effects of combined therapies, whereas the same effect was not corroborated with radiation alone. On the other hand, tumours in mice treated with cetuximab exhibited a complete abrogation in the levels of phosphorylation of EGFR and ERK even when the antibody was administered as a single agent. These results indicate a higher capacity to block EGFR signalling in tumour cells for cetuximab, compared with nimotuzumab, accordingly with earlier findings (Diaz Miqueli *et al*, 2007). Moreover, the different treatments did not produce significant changes in the endogenous levels of ERK proteins (Figure 5, bottom panel).

### Targeting of CD133+ radioresistant cancer stem cell in U87MG tumours *in vivo* by anti-EGFR mAb

**Figure 6:**
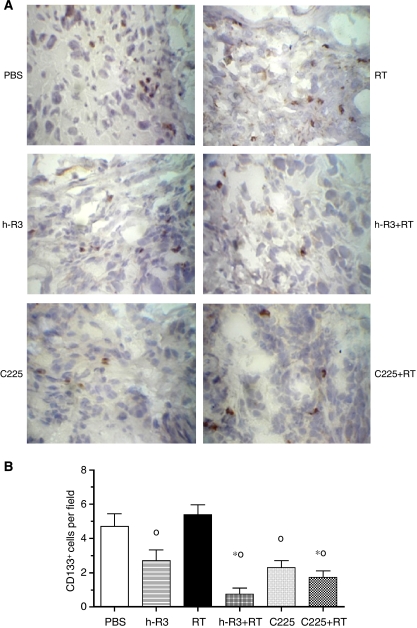
Tissue-based studies of U87MG human tumours xenografted into NMRI nude mice treated with nimotuzumab (h-R3), or cetuximab (C225), or radiation alone (RT), or both modalities, or neither (PBS). (**A**) Cryostat sections were fixed and immunostained with CD133/1 (AC133) antibody yielding a staining pattern typical for membrane ( × 40 magnification). (**B**) Comparisons between groups. Kruskal–Wallis test; symbols indicate statistical differences (*P*<0.05) as follows: ^*^Significant to PBS, °significant to radiation.

Brain tumour growth has recently postulated being critically dependent on the presence of an intact cancer stem cell (CSC) vascular niche, representing a potential target for treatments of brain tumours (Calabrese *et al*, 2007). We therefore investigated whether anti-EGFR mAb might reduce the number of CSC from this brain tumour. As glioma subpopulations expressing Prominin-1 (CD133+) are enriched for CSC, which mark quite specifically brain CSC, we quantified the expression of CD133+ cells in U87MG tumours. As shown in Figures 6A and B, co-administration of both antibodies with radiation was associated to a significant reduction in the total number of CD133+ cells in analysed tumours specimens. The frequency of CD133+ cells per field (mean±s.e.m.) decreased from 4.7±0.8 in control mice to 0.8±0.4 and 1.7±0.4 in mice receiving h-R3+RT and C225+RT, respectively. Coincidently to the effect observed on satellite tumours, animals treated with h-R3 or C225 mAb as monotherapies, displayed a significant reduction in the frequency of CD133+ cells per field (2.7±0.6 and 2.3±0.4, respectively), compared with radiotherapy (5.4±0.6).

Radiation alone resulted in slightly non-significant increases in the percentages of CD133+ cells relative to untreated mice, in line with earlier reports obtained in cultures from human glioma xenografts (Bao *et al*, 2006). These results may suggest that anti-EGFR mABs may radiosensitisate U87MG brain tumours.

## Discussion

Despite aggressive therapies, malignant gliomas remains resistant to all currently used conventional treatment modalities, such as radiation and chemotherapy. Current research focuses on immunotherapy as a novel approach of targeted molecular therapies on glioma treatment, on the basis of the presence of potential antigens such as EGFR (Barker *et al*, 2001). Radiosensitisation of tumour cell mediated by EGFR antagonists is supported by preclinical and clinical studies (Bonner *et al*, 2006; Ramos *et al*, 2006), also suggesting the possibility of successful radiosensitisation in this setting. To better understand the influence of the EGFR blockade on radiation response in GBM, we investigated the antitumour effect of combined treatment with two mAb to EGFR and radiation in human tumour U87MG xenografts.

The antitumour effect of anti-EGFR mAbs in combination with radiotherapy has been thought to result from the enhancement of the inhibition of EGFR signalling, increasing the cytotoxic effect of the radiation (Akashi *et al*, 2008). Consistent with these results, we found that treatment with nimotuzumab and radiation enhanced the inhibition of EGFR-signalling activation, supporting a notion that the inhibition of ERK-mediated signalling is related to radiation response *in vivo* in GBM (Xia *et al*, 1995). Such inhibition was not apparent for tumours treated with radiation alone, suggesting a rationale for combined treatment with anti-EGFR mAbs and radiotherapy. Contrastingly, no antitumor activity was observed in mice treated with cetuximab alone, despite inhibiting completely EGFR signalling, suggesting that different mechanisms of action of the two antibodies underwent the enhancement of the cytotoxic effects of radiation. Whereas cetuximab seems to affect the tumour cells directly, by abrogating EGFR activation, nimotuzumab would target both endothelial cells and tumour cells through a significant anti-angiogenic activity. Nimotuzumab was shown earlier to induce the regression of A431 tumour xenografts *in vivo* as a result of a potent antiangiogenic activity. One of the endothelial-specific growth factors often associated with tumour angiogenesis is vascular endothelial growth factor (VEGF) (Johnson *et al*, 2007). Further, a close relationship between EGFR/VEGF has been postulated to promote angiogenesis (Rak *et al*, 2000). Whereas earlier studies indicated that VEGF is induced in tumours after irradiation (Gorski *et al*, 1999), nimotuzumab has shown to downregulate VEGF expression at the RNA and protein level after treatment in A431 tumour xenografts *in vivo* (Crombet-Ramos *et al*, 2002). In agreement with that, we found a 60% of reduction in the total area of the vessels in tumour specimens from mice treated with nimotuzumab. Whether a two-fold reduction in the area of the vessels would translate into a significant antitumour activity remains unclear, suppression of VEGF expression in a GBM cell line, in the order of three-fold, has reported to abrogate its growth in nude mice (Cheng *et al*, 1996). In line with these considerations, the targeting of the vascular microenvironment in brain tumours to arrest tumour growth has been proposed to explain mechanisms associated to drugs with antiangiogenic potential (Calabrese *et al*, 2007). These observations suggest that an antiangiogenic and targeted therapy against both endothelial cells and tumour cells may inhibit brain tumour growth by disrupting the vascular endothelial microenvironment, in addition to abrogate the EGFR activation.

Interestingly, CSC has been identified as a new cellular subpopulation of brain tumour cells with potent tumourigenic activity (Hemmati *et al*, 2003; Singh *et al*, 2003). As glioma subpopulations CD133+ show greater tumourigenic potential than do CD133− cells, an important role in the development of glioma radioresistance has been conferred to these cells (Bao *et al*, 2006). A recent investigation has showed a close relationship between endothelial cells and these brain CSCs resulting in the initiation and growth of tumours (Calabrese *et al*, 2007). Therefore, ongoing investigation has linked the therapeutic activity of antiangiogenic therapies against GBM to their intrinsic capacity to deplete tumour blood vessels and CSC. These findings might support the use of tumour microvasculature targeted therapies in the design of future therapeutic approaches against brain tumours. Despite future investigations are needed to elucidate the role of anti-EGFR agents on specific CD133+ CSC, this study has first suggested the feasibility of targeting this radioresistant cell subpopulation by using specific anti-EGFR mAbs against GBM.

A question emerging from this study was the lack of therapeutic response when nimotuzumab was given alone, despite shown a significant antiangiogenic activity. The reason is not fully understood, but it is likely that a combination of different mechanisms is needed to translate into a relevant therapeutic response. Indeed, the combined treatment with nimotuzumab and radiotherapy enhanced the antiproliferative activity of both drugs compared with individual therapies. Similarly, the absence of activity of cetuximab given alone, despite the abrogation of the EGFR signalling, suggest that it is at least in part responsible for the enhancement of the cytotoxic effects of radiation, but not enough to produce a substantial therapeutic response.

More important, both antibodies displayed capacity to inhibit the tumour cell invasion provoked by the radiotherapy. It has established that the blockade of angiogenesis leads to compensatory mechanisms by which tumours may recruit their own vascular supply from pre-existent vessels and grow despite the absence of neovascularisation, a phenomenon named as vessel co-option (Holash *et al*, 1999). Furthermore, EGFR signalling can strongly stimulate GBM cell migration and invasion (Lund-Johansen *et al*, 1990). In line with these findings, Cetuximab has shown to inhibit the increased glioma cell invasion after the therapy with DC101, a mAb against VEGFR-2 with a potent antiangiogenic activity (Lamszus *et al*, 2005). In sum, these data point out that the success of either ionising radiation or antiangiogenic therapies may increase by simultaneous blockade of EGFR.

Glioma invasiveness has been linked to the development of local tumour satellites modulated after therapeutic interventions (Lamszus *et al*, 2005). That observation was corroborated in our experimental conditions (Figure 2B and Supplementary Table 1). A current paradigm postulates that irradiation in a dose-dependent manner promotes migration and invasiveness of glioma cells (Wild-Bode *et al*, 2001). This effect involves remodelling and the growth of experimental tumours (Brooks *et al*, 1996; Silletti *et al*, 2001; Wild-Bode *et al*, 2001; Wick *et al*, 2002). The recent demonstration that irradiation increases circulating levels of TGF-*β*, circulating cancer cells and tumour metastases by a direct effect of the TGF-*β* on the cancer cells may also contribute to explain that unexpected biological effect of radiotherapy (Biswas *et al*, 2007).

Furthermore, the possible interrelations of TGF-*β*, proteins of the BCL-2 family, integrins and metalloprotease activity may promote glioma invasion (Wick *et al*, 2001). Others have suggested that TGF-*β* could mediate EGFR transactivation, and subsequently activate ERK and p38MAPK through PKC (Uchiyama-Tanaka *et al*, 2002).

In addition, radiation therapy may enhance the EGFR intracellular activation pathways after irradiation, which in turn may contribute to enhance the tumour invasiveness (Figure 5). EGFR in the context of the human gliomas results in a strong stimulator of tumour cell migration and invasion (Lund-Johansen *et al*, 1990; Penar *et al*, 1997; Lamszus *et al*, 2005). Despite more investigation is needed, these evidences suggest that the blockade of the EGFR by specific mAbs may antagonise the role of the TGF-*β* on promote tumour cell invasion and metastasis after a radiation therapy.

In summary, we have provided evidence that nimotuzumab and cetuximab, two specifics mAb to EGFR, may increase the radiosensitivity of human U87MG tumour, enhancing the antitumour efficacy of radiation *in vivo.* This work may enhance our preclinical understanding of the interaction of radiation and the inhibition of the EGFR to increase radiation-induced tumour toxicity. This information further serves as preclinical background for clinical investigations involving anti-EGFR antibodies with radiation in human GBM, a tumour relatively resistant to the treatment with other well known anti-EGFR agents, such as Iressa and Tarceva (Newton, 2003; Rich *et al*, 2004).
