# Supplementary figures and images for: Radiosensitisation of U87MG brain tumours by anti-epidermal growth factor receptor monoclonal antibodies
Source: Br J Cancer. 2009 Mar 17;100(6):950–8. doi: 10.1038/sj.bjc.6604943 (PMC2661790; doi:10.1038/sj.bjc.6604943)

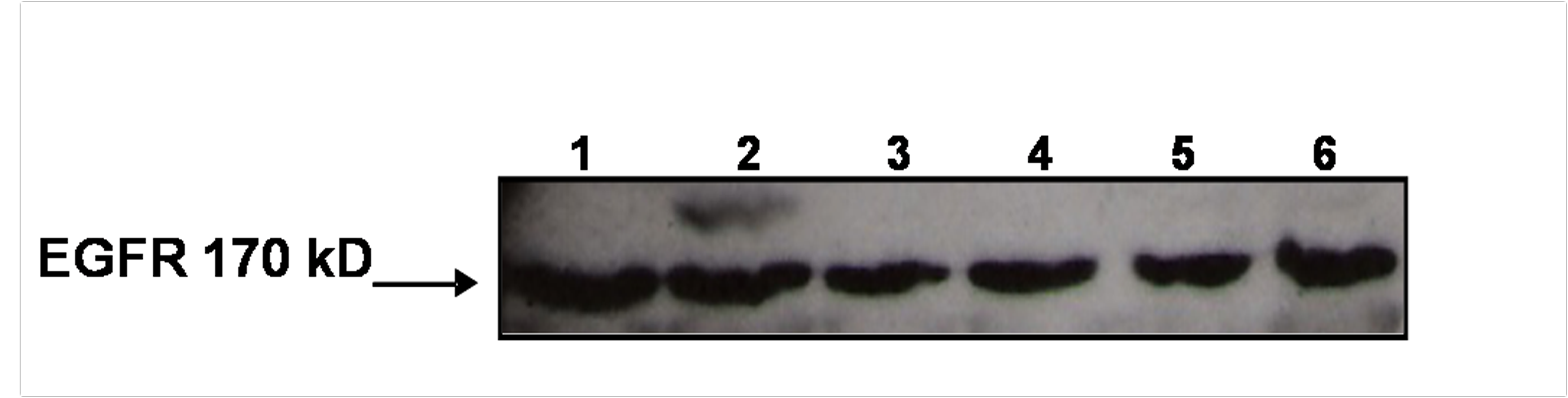

Supplement: Supplementary Figure 1 [file 6604943x1.tif]
