# Supplementary material for: Radiosensitisation of U87MG brain tumours by anti-epidermal growth factor receptor monoclonal antibodies
Source: Br J Cancer. 2009 Mar 17;100(6):950–8. doi: 10.1038/sj.bjc.6604943 (PMC2661790; doi:10.1038/sj.bjc.6604943)
Supplement: Supplementary Information [file 6604943x3.doc]

SUPPLEMENTARY INFORMATION

**Supplementary Table 1** Satellite tumour formation in the brain of NMRI nude mice injected intracranial with 10^4^ U87MG cells and treated with nimotuzumab (h-R3), cetuximab (C225), radiation alone (RT), or both modalities. Values represent minimum, maximum and the median of total satellite tumours in each group. The percent of change is referred to control group.

**Supplementary Figure 1** Tissue-based studies of U87MG human tumours xenografted into NMRI nude mice treated with nimotuzumab (h-R3), or cetuximab (C225), or radiation alone (RT), or both modalities. Immunoblot analysis of tumour cells stained with anti-EGFR antibody. Lane 1 PBS control; lane 2 nimotuzumab; lane 3 radiation; lane 4 nimotuzumab plus radiation; lane 5 cetuximab; lane 6 cetuximab plus radiation. Immunoblots were developed using chemiluminescence. Pictures show one representative example per group out of 4 samples analysed.
